# *Staphylococcus aureus* aggregation in the plasma fraction of silkworm hemolymph

**DOI:** 10.1371/journal.pone.0217517

**Published:** 2019-05-30

**Authors:** Hiroki Ryuno, Fuki Nigo, Isao Naguro, Kazuhisa Sekimizu, Chikara Kaito

**Affiliations:** 1 Graduate School of Pharmaceutical Sciences, The University of Tokyo, Tokyo, Japan; 2 Graduate School of Medicine, Dentistry, and Pharmaceutical Sciences, Okayama University, Okayama, Japan; 3 Institute of Medical Mycology, Teikyo University, Tokyo, Japan; Institute of Plant Physiology and Ecology Shanghai Institutes for Biological Sciences, CHINA

## Abstract

*Staphylococcus aureus* formed bacterial aggregates in the plasma fraction of the hemolymph of silkworm, the larva of *Bombyx mori*, in a growth-dependent manner. The addition of arabinose or galactose inhibited the formation of *S*. *aureus* aggregates in the silkworm plasma. Formation of the bacterial aggregates depended on *S*. *aureus* genes required for the synthesis of bacterial surface polysaccharides–*ypfP* and *ltaA*, which are involved in lipoteichoic acid synthesis, and the *tagO* gene, which is involved in wall teichoic acid synthesis. These findings suggest that *S*. *aureus* forms bacterial aggregates in the silkworm plasma *via* bacterial surface teichoic acids.

## Introduction

*Staphylococcus aureus* is a human pathogenic bacterium that exists in the nares of 30% healthy persons and causes various diseases such as pneumonia, meningitis, and sepsis in immunocompromised patients. Since the 1960s, methicillin-resistant *S*. *aureus* (MRSA) has infected many people in hospitals and clinical care facilities, and a new type of MRSA, called community-acquired MRSA, has emerged in the last two decades, infecting healthy persons in the general population [1]. Overcoming *S*. *aureus* infectious diseases will require a comprehensive understanding of the detailed molecular mechanisms underlying *S*. *aureus* virulence.

*S*. *aureus* forms aggregates in mammalian blood plasma, which is called the “coagulase reaction” [2]. The coagulase reaction is an important clinical feature for identifying *S*. *aureus* [3–5]. *S*. *aureus* aggregation caused by the coagulase reaction facilitates bacterial escape from host immune responses and contributes to *S*. *aureus* virulence [6–8]. Thus, clarification of the molecular mechanism of the aggregation reaction of *S*. *aureus* in animal blood plasma is important for understanding *S*. *aureus* virulence. Although many molecular aspects of the coagulase reaction in mammalian plasma have been studied, it has remained unclear whether *S*. *aureus* forms aggregates in the plasma fraction from animals other than mammals.

Animal infection models are essential for investigating the virulence mechanism of human pathogenic bacteria, but the use of mammals such as mice is limited due to ethical and economical issues. On the other hand, large numbers of invertebrate animals such as silkworms, fruit flies, and nematodes, which share similar innate immune systems with mammals, can be used more easily, and have been used as animal models of human pathogenic bacterial infection [9, 10]. We have used silkworms to investigate the virulence system of *S*. *aureus* and the host innate immune system against *S*. *aureus* [9, 11]. *S*. *aureus* novel virulence factors identified by using a silkworm infection model contribute to *S*. *aureus* virulence in mice [12–15]. Lipid carrier proteins of both silkworms and mice suppress *S*. *aureus* virulence [16–18]. These findings suggest the usefulness of the silkworm infection model to investigate *S*. *aureus-*host interactions [19]. In the present study, we investigated whether *S*. *aureus* forms aggregates in silkworm plasma. We observed *S*. *aureus* aggregation in silkworm plasma and demonstrated the requirement of *S*. *aureus* genes involved in synthesis of teichoic acid, an *S*. *aureus* cell surface polysaccharide, for *S*. *aureus* aggregation.

## Materials and methods

### Bacterial strains and culture conditions

*S*. *aureus* was aerobically cultured in tryptic soy broth (TSB) at 37°C using an air shaker at a speed of 150 rpm (BR-3000LF, TAITEC, Tokyo, Japan). To culture *S*. *aureus* gene knockout strains, TSB was supplemented with 10 μg/ml erythromycin. The bacterial strains and plasmids used in this study are shown in Table 1.

**Table 1:** List of bacterial strains and plasmids used.

Strain or plasmid	Genotypes or characteristics	Source or reference
Strains		
*S*. *aureus*		
RN4220	NCTC8325-4, restriction mutant	[41]
M0875	RN4220 Δ*ypfP*::pT0875R	This study
M0874	RN4220 Δ*ltaA*::pT0874	This study
M0702	RN4220 Δ*tagO*::pT0702	[42]
MSSA1	Methicillin-sensitive clinical isolate	[43]
NCTC8325-4	NCTC8325 cured of ϕ11, ϕ12, and ϕ13	[41]
COL	Methicillin-resistant clinical isolate	[44]
Plasmids		
pMutinT3	Suicide vector for gene-disruption; Amp^r^, Erm^r^	[45]
pT0875R	pMutinT3 with partial *ypfP* gene from RN4220	This study
pT0874	pMutinT3 with partial *ltaA* gene from RN4220	This study
pT0702	pMutinT3 with partial *tagO* gene from RN4220	[42]
pHY300	*E*. *coli*-*S*. *aureus* shuttle vector; Amp^r^ Tet^r^	Takara Bio
pypfP	pHY300 with intact *ypfP* from RN4220	[42]
pltaA	pHY300 with intact *ltaA* from RN4220	This study
ptagO	pHY300 with intact *tagO* from RN4220	[42]

Amp: ampicillin, Erm: erythromycin, Cm: chloramphenicol, Tet: tetracyclin.

### Construction of *S*. *aureus* gene knockout strains

*S*. *aureus* gene-knockout mutant was constructed as we previously reported with minor modifications [12]. An internal region of the *ypfP* or *ltaA* gene was amplified by polymerase chain reaction (PCR) from the RN4220 genome as a template using oligonucleotide primers (Table 2). The amplified DNA fragment was inserted into pMutinT3, resulting in pT0875R or pT0874. *S*. *aureus* RN4220 strain was electroporated with pT0875R or pT0874 by using an electroporator (Gene Pulser Xcell, BIO-RAD) in 0.2 cm cuvette at 2.3 kV, 25 microfarads, and 100 Ω, of which condition was slightly modified from our previous method [20]. The electroporated cells was immediately suspended in B2 broth (Casamino acids, 10g/l; Yeast extract, 25g/l; K_2_HPO_4_, 1g/l; Glucose, 5g/l; NaCl, 25g/l)[21] and incubated for 1 h at 37°C. The cells were spread on TSB agar plates containing 10 μg/ml erythromycin and the plates were incubated at 37°C for more than 2 days and colonies were obtained. The single colony was cultured overnight in TSB medium supplemented with 10 μg/ml erythromycin at 37°C and the proliferated bacterial cells were treated with 10 μg/ml lysostaphin. The genomic DNA was extracted as reported previously [22]. The gene knockout was confirmed by Southern blot analysis.

**Table 2:** PCR primers used in the study.

Target	Primer	Sequence (5'-3')
*ypfP*	ypfP-F	AAGAAGCTTTTACAGCCGCCCAGATAAAC
	ypfP-R	GGAGGATCCCCAGGTGCAGGATTTAGGAA
*ltaA*	ltaA-F	AAGAAGCTTAAAATTCGGCACAAAAATCG
	ltaA-R	GGAGGATCCTAGCATCGAAACTGCACAGC
*ltaA* (complementation)	ypfPltaAcomp-F	TCCAACTGAAGCGACAAAAA
	ypfPltaAcomp-R	CGTTTTGACGATGACGAAGA
	ypfPdel-F	CGGTCATTCATCACAACCAC
	ypfPdel-R	ATGACCGTTACCGAATGAGC

### Construction of a plasmid carrying the *ltaA* gene

To construct pltaA containing the *ltaA* gene with the native promoter, DNA fragment containing the native promoter and the *ypfP-ltaA* operon was amplified by PCR from the RN4220 genome using oligonucleotide primer pairs (ypfPltaAcomp-F and ypfPltaAcomp-R) and KOD plus DNA polymerase (TOYOBO co. ltd., Osaka, Japan) (Table 2). The amplified DNA fragment was inserted into pHY300, resulting in pypfP-ltaA. To remove the *ypfP* region from pypfP-ltaA, DNA fragment without the *ypfP* region was amplified by PCR from pypfP-ltaA using ologonucleotide primer pairs (ypfPdel-F and ypfPdel-R) (Table 2) and self-ligated, resulting in pltaA.

### Preparation of silkworm plasma

Fertilized eggs of silkworms (Hu/Yo × Tsukuba/Ne) were purchased from Ehime Sansyu (Ehime, Japan). Hatched silkworms were raised in a laboratory as previously described [15, 23]. Prolegs of fifth instar silkworms at day 2 after molting were cut and the dropped hemolymph was collected into ice-cold 50 mL conical tubes (Falcon) using a funnel. The collected hemolymph was supplemented with 0.05 mM phenylthiourea and centrifuged at 8,600 *g* at 4°C for 10 min. The centrifuged supernatant was frozen in liquid nitrogen and stored at -80°C. The frozen supernatant was melted and used as silkworm plasma.

### *S*. *aureus* aggregation in silkworm plasma

A single colony of *S*. *aureus* was aerobically cultured in TSB at 37°C overnight. The overnight culture (50 μl) was inoculated into silkworm plasma (2 ml) in 50 mL conical tubes (Falcon), and aerobically cultured at 37°C at a speed of 150 rpm. After 7 h culturing, aggregates were collected by centrifugation at 70 *g* at room temperature for 5 min (RL-131, TOMY SEIKO CO., Tokyo, Japan). The aggregates were suspended with 2 ml of phosphate buffered saline (PBS) and centrifuged at 70 *g* at room temperature for 5 min. The washing was repeated one more time. The aggregates were suspended in 1 ml PBS and poured into 10 x 35 mm dishes (Corning). The dishes were photographed by using a scanner (LiDE 210, CANON Inc., Tokyo, Japan). To measure the amount of the aggregates, the aggregates were dried in 1.5 ml tubes using a centrifuge evaporator (CC-105, TOMY SEIKO CO., Tokyo, Japan) and the dry weight was measured. We used 7 h cultured sample for measurement of bacterial aggregates, because enough bacterial aggregates were observed at 7 h culturing.

### Microscopy of *S*. *aureus* aggregates

*S*. *aureus* aggregates were suspended in PBS, placed on slide glass, and Gram-stained (Merck). The slide glass was observed under a light microscope (DM4000B, Leica).

### Requirement of bacterial growth for *S*. *aureus* aggregation in silkworm plasma

A single *S*. *aureus* colony was aerobically cultured in TSB at 37°C overnight. The *S*. *aureus* overnight culture was autoclaved at 121°C for 15 min. The autoclaved or non-autoclaved culture (2 ml) was centrifuged at 21,400 *g* at 4°C for 5 min, and the precipitated *S*. *aureus* cells were suspended in 1 ml of PBS. The cells were collected by centrifugation at 21,400 *g* at 4°C for 5 min, and were used as heat-killed cells or viable cells, respectively. The heat-killed or viable *S*. *aureus* cells were suspended in 2 ml of silkworm plasma, and were aerobically incubated at 37°C using an air shaker at a speed of 150 rpm. After 7 h incubation, the aggregates were analyzed as described above.

### Examination of the inhibitory effect of monosaccharides on *S*. *aureus* aggregation in silkworm plasma

*S*. *aureus* overnight culture (50 μl) was inoculated into 2 ml of silkworm plasma supplemented with 200 mM of monosaccharide (glucose, galactose, mannose, maltose, arabinose, or N-acetyl-glucosamine) and cultured at 37°C using an air shaker at a speed of 150 rpm. After 7 h culturing, the aggregates were analyzed as described above.

### Silkworm infection experiment

A single colony of *S*. *aureus* was aerobically cultured in TSB at 37°C overnight. The *S*. *aureus* overnight culture was serially diluted with PBS. To concentrate the bacterial solution, the overnight culture was centrifuged at 8,600 *g* at 4°C for 5 min, and bacterial cell pellet was suspended in an appropriate amount of PBS. Fifth instar silkworms were fed an antibiotic-free artificial diet (Katakura Industries, Tokyo, Japan) for 1 day, and were injected with bacterial solution via the intra-hemolymph route. Silkworm survival was examined at 24 h after the bacterial injection. Because the *S*. *aureus* gene knockout strains used in this study produced fewer colony forming units than the parent strain, we performed an ATP-based assay to measure the viable bacterial cell number, as described previously [24]. Briefly, the ATP concentration in the *S*. *aureus* overnight culture was measured using firefly luciferase and the substrate (Kikkoman Corp., Tokyo, Japan), and *S*. *aureus* viable cell number per culture volume was calculated. To determine the half-maximal lethal dose (LD_50_), the survival curve was determined by logistic regression based on the dose-response survival plots [25].

## Results

### *S*. *aureus* forms bacterial aggregates in silkworm plasma

**Fig 1:**
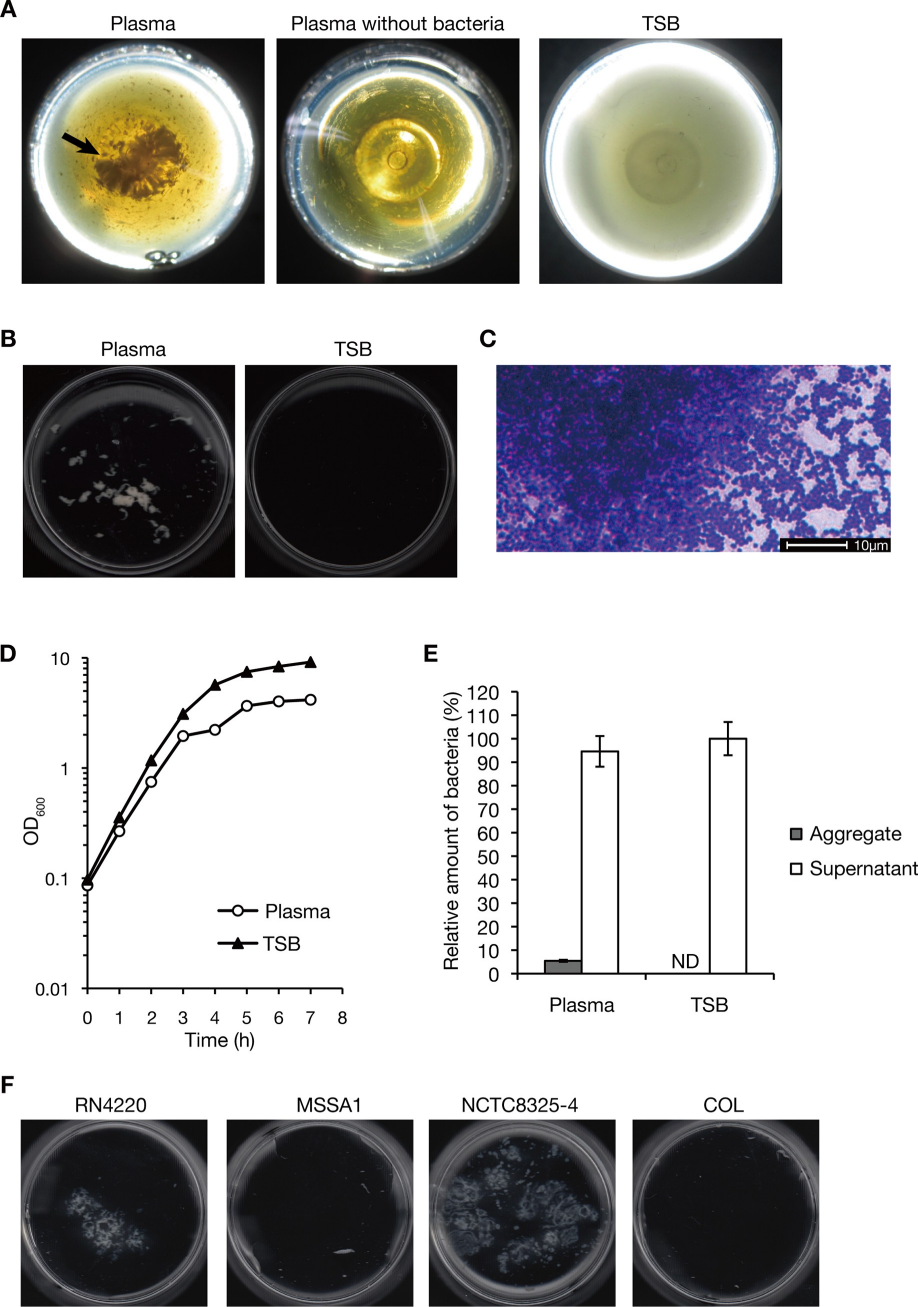
*S*. *aureus* forms bacterial aggregates in silkworm plasma. *A) S*. *aureus* overnight culture (0.05 ml, 1.5 x 10^8^ CFU) was inoculated into 2 ml of silkworm plasma (left) or TSB (right) and was incubated at 37°C for 7 h. Silkworm plasma without bacterial inoculation was incubated in the same condition (center). Precipitate in silkworm plasma is indicated by a black arrow. *B) S*. *aureus* was cultured in silkworm plasma or TSB at 37°C for 7 h. The culture was centrifuged at 70 *g* for 5 min to collect precipitate and the precipitate was spread in a Petri dish. *C) S*. *aureus* was cultured in silkworm plasma at 37°C for 7 h. The formed precipitate were Gram-stained and examined under a light microscope. D) *S*. *aureus* growth curves in silkworm plasma or TSB at 37°C were measured. A fluid part of bacterial culture was sampled during the time course of incubation and the OD_600_ value was measured. E) Relative weights of the bacterial aggregates and the planktonic bacteria to the total bacterial weight were measured. *S*. *aureus* was cultured in silkworm plasma or TSB at 37°C for 7h. The bacterial aggregates were collected by low centrifugation and the dry weight of the aggregates was measured. The planktonic bacterial cells in the centrifuged supernatant were collected by further centrifugation at 21,400 *g* for 5 min and the dry weight was measured. Vertical axis represents the relative weight to the total bacterial weight in plasma or TSB. ND means not detected. *F) S*. *aureus* strains including RN4220, MSSA1, NCTC8325-4, and COL were cultured in silkworm plasma at 37°C for 7 h. Bacterial aggregates were spread in a Petri dish.

Overnight culture of *S*. *aureus* RN4220 strain was inoculated into silkworm plasma and aerobically cultured at 37°C. After 7 h incubation, precipitates were observed at the bottom of the culture tube (Fig 1A). The precipitates were not observed when *S*. *aureus* was cultured in TSB or *S*. *aureus* was not inoculated into silkworm plasma (Fig 1A). The precipitates were collected by low centrifugation (70 *g* for 5 min), whereas the planktonic *S*. *aureus* cells in TSB was not collected by this centrifugation condition (Fig 1B). The precipitates were Gram-stained and analyzed under a microscope. The precipitates contained many numbers of *S*. *aureus* cells (Fig 1C), suggesting that *S*. *aureus* forms bacterial aggregates in silkworm plasma. In the beginning of incubation, the bacterial growth curves were not much different between plasma and TSB, but in the later stage of incubation, *S*. *aureus* growth in silkworm plasma was decreased compared to that in TSB (Fig 1D), when the bacterial aggregates were observed. The weight of bacterial aggregates was 5% of the total bacterial weight (Fig 1E).

We examined whether the *S*. *aureus* aggregation activity was observed in other *S*. *aureus* strains. *S*. *aureus* NCTC8325-4 strain formed a large amount of aggregates, whereas *S*. *aureus* MSSA1 and COL strains formed few aggregates (Fig 1F). The result indicates that the aggregation activities are different among *S*. *aureus* strains. We used *S*. *aureus* RN4220 strain for the subsequent analysis of this study.

### *S*. *aureus* aggregation in silkworm plasma requires bacterial growth

**Fig 2:**
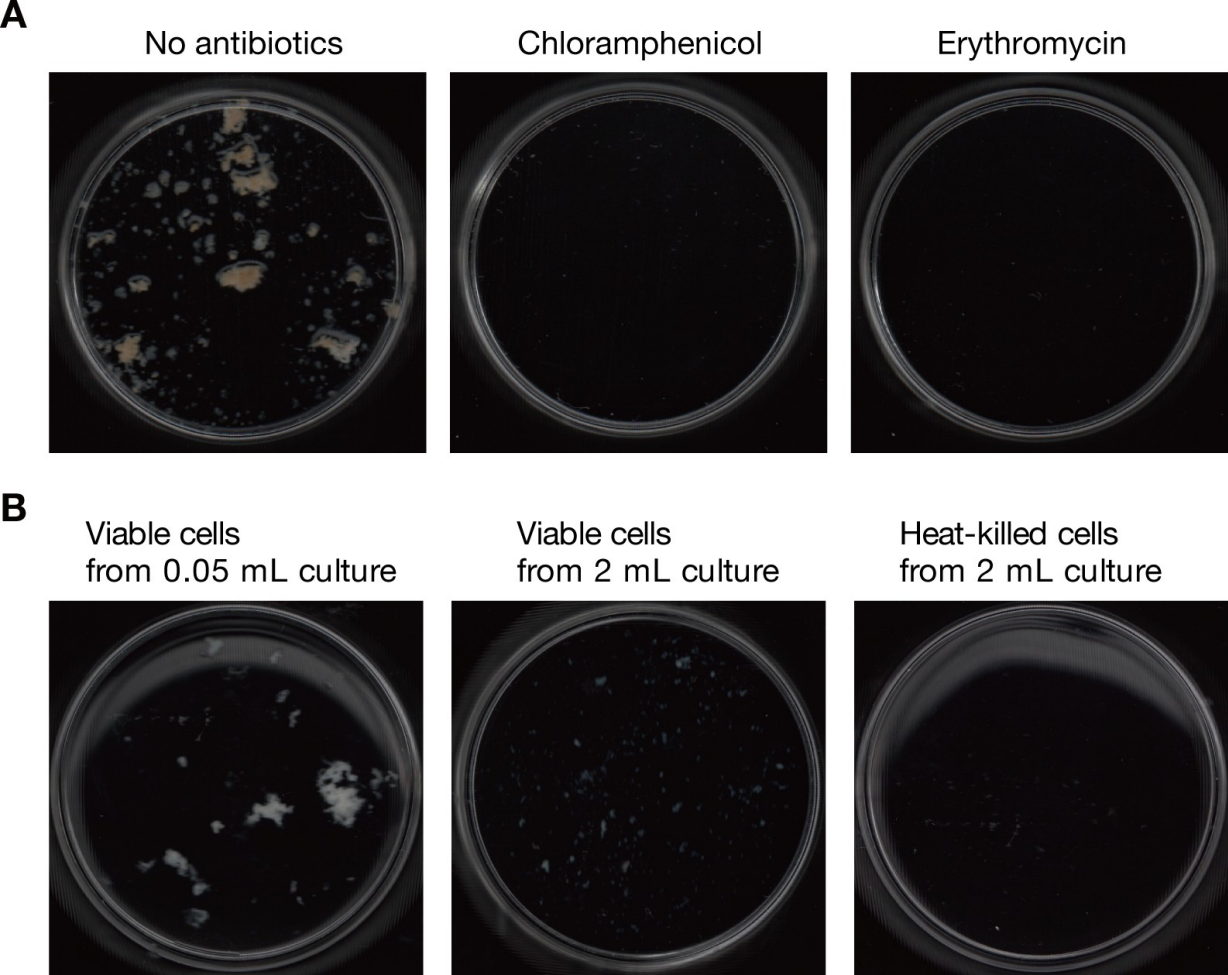
*S*. *aureus* growth is required for *S*. *aureus* aggregation in silkworm plasma. *A) S*. *aureus* was cultured in silkworm plasma or in silkworm plasma supplemented with 50 μg/ml chloramphenicol or 10 μg/ml erythromycin at 37°C for 7 h. The aggregates were collected and spread in a Petri dish. *B) S*. *aureus* viable cells from 0.05 mL overnight culture (left), *S*. *aureus* viable cells from 2 mL overnight culture (center), or *S*. *aureus* heat-killed cells from 2 mL overnight culture (right) was inoculated into 2 ml of silkworm plasma, and cultured at 37°C for 7 h. The aggregates were collected and spread in a Petri dish.

To understand the molecular mechanism for *S*. *aureus* aggregation in silkworm plasma, we examined whether *S*. *aureus* growth is required for the formation of *S*. *aureus* aggregates in the silkworm plasma. *S*. *aureus* cells were inoculated into silkworm plasma with or without antibiotics (chloramphenicol or erythromycin) and incubated for 7 h. The addition of antibiotics completely abolished the *S*. *aureus* aggregation in silkworm plasma (Fig 2A). To further address whether bacterial growth or presence of enough number of viable cells are required for the *S*. *aureus* aggregation, we examined the aggregation capacities of heat-killed *S*. *aureus* cells or *S*. *aureus* viable cells in silkworm plasma, in which cell concentration is corresponding to that at full growth phase. Viable *S*. *aureus* cells from 2 mL overnight culture or heat-killed *S*. *aureus* cells from 2 mL overnight culture was inoculated into 2 mL of silkworm plasma, and aerobically cultured at 37°C. After 7 h incubation, both the viable cells from 2 mL culture and the heat-killed cells severely decreased the formation of the bacterial aggregates (Fig 2B). On the other hand, viable *S*. *aureus* cells from 0.05 mL overnight culture did produce bacterial aggregates (Fig 2B). These findings suggest that *S*. *aureus* growth in silkworm plasma is required for *S*. *aureus* aggregation in silkworm plasma.

### *S*. *aureus* aggregation in silkworm plasma is inhibited by the addition of monosaccharides

**Fig 3:**
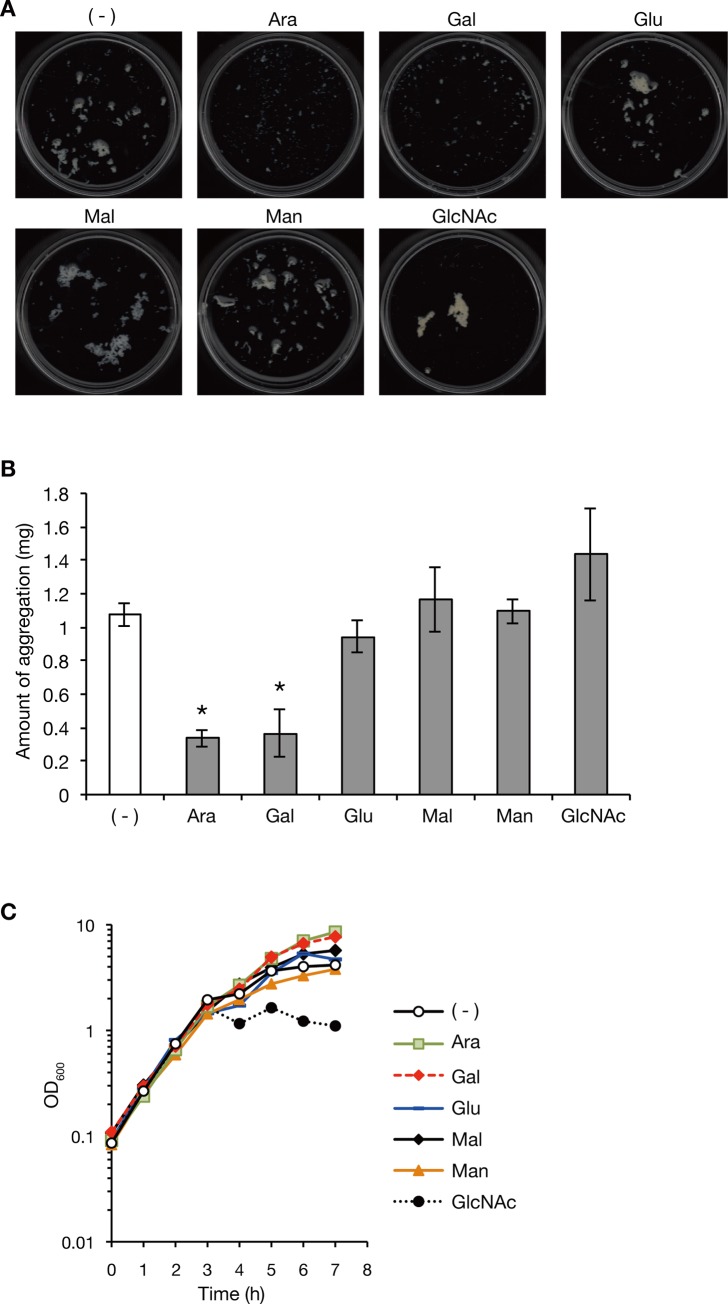
*S*. *aureus* aggregation in silkworm plasma is inhibited by the addition of monosaccharides. A) *S*. *aureus* was cultured in silkworm plasma supplemented with or without monosaccharides [arabinose (Ara), galactose (Gal), glucose (Glu), maltose (Mal), mannose (Man), or N-acetyl-glucosamine (GlcNAc)] at 37°C for 7 h. Bacterial aggregates were spread in a Petri dish. B) The dry weight of the aggregates observed in A) was measured. Means±standard errors from three independent experiments are presented. Asterisks indicate Student’s t-test p-value less than 0.05 between the absence and presence of monosaccharide. C) *S*. *aureus* growth curves in silkworm plasma supplemented with or without monosaccharides [arabinose (Ara), galactose (Gal), glucose (Glu), maltose (Mal), mannose (Man), or N-acetyl-glucosamine (GlcNAc)] were measured at 37°C. This experiment was performed in parallel with the experiment of Fig 1D and the growth curve in silkworm plasma without monosaccharide is same with the data in Fig 1D (Plasma).

Lectin binds to polysaccharides of foreign substances and functions in host defense. Monosaccharides inhibit lectin binding [26]. We examined whether the addition of monosaccharides inhibits *S*. *aureus* aggregation in silkworm plasma. *S*. *aureus* was aerobically cultured in silkworm plasma supplemented with or without several monosaccharides (arabinose, galactose, glucose, maltose, mannose, and N-acetyl-glucosamine) at 37°C. Addition of arabinose and galactose decreased the amount of *S*. *aureus* aggregates in silkworm plasma, whereas glucose, maltose, mannose, and N-acetyl-glucosamine did not decrease the amount of *S*. *aureus* aggregates (Fig 3A and 3B). We measured the *S*. *aureus* growth curves in silkworm plasma supplemented with these monosaccharides to see whether the effect of monosaccharide is due to bacterial growth inhibition. The addition of all of these monosaccharides did not inhibit bacterial growth during 0–3 h (Fig 3C). There were differences of growth curves during 4–7 h, in which bacterial growth in the presence of arabinose and galactose were higher than that without addition of monosaccharide, whereas bacterial growth in the presence of N-acetyl-glucosamine was lower than that without addition of monosaccharide (Fig 3C). These results suggest that arabinose and galactose inhibit *S*. *aureus* aggregation without inhibiting bacterial growth.

### *S*. *aureus* teichoic acids are required for *S*. *aureus* aggregation in silkworm plasma

**Fig 4:**
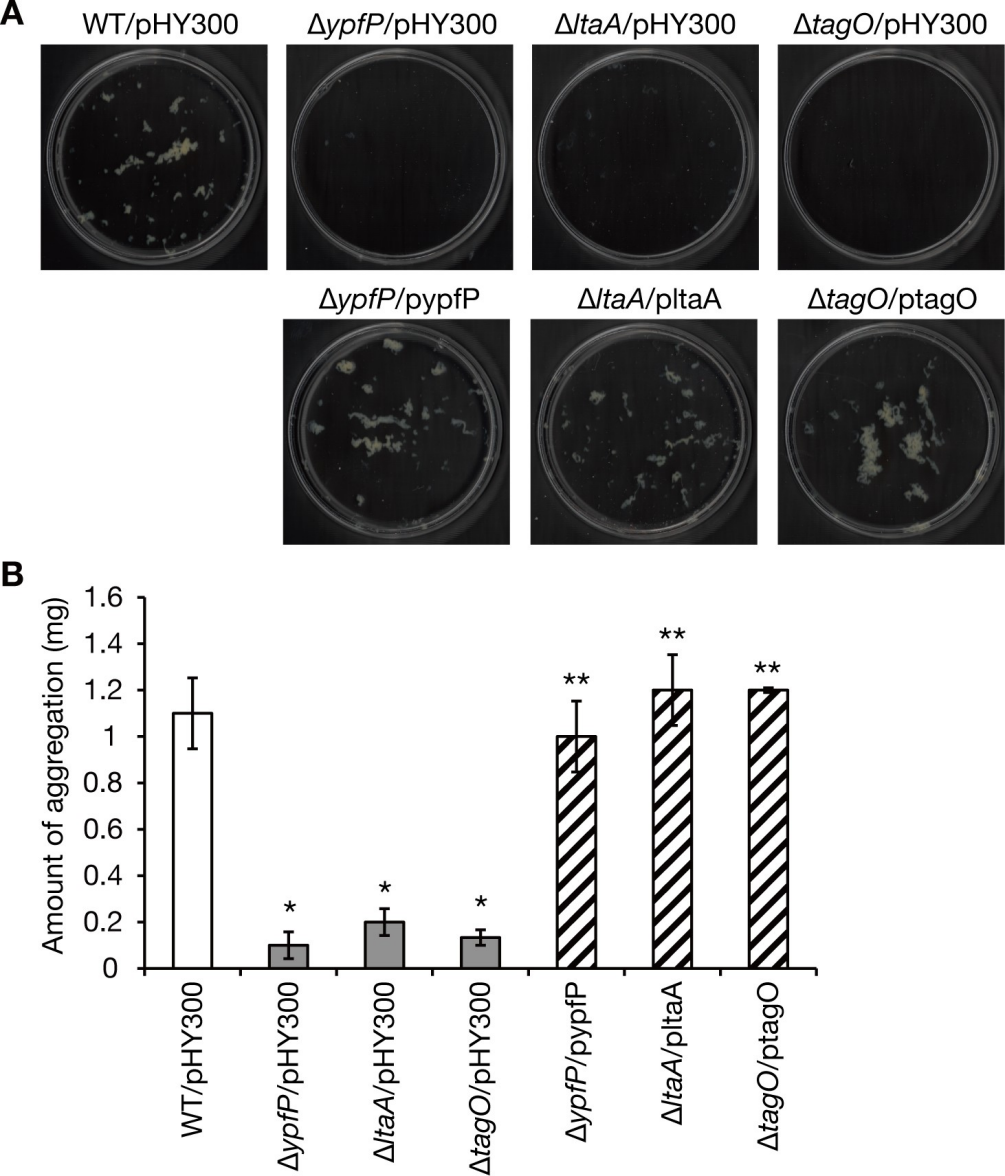
Knockout of the *ypfP*, *ltaA*, and *tagO* genes decreases *S*. *aureus* aggregation in silkworm plasma. A) The *S*. *aureus* parent strain (WT) transformed with an empty vector (pHY300) and knockout strains of the *ypfP*, *ltaA*, and *tagO* genes (Δ*ypfP*, Δ*ltaA*, Δ*tagO*), which were transformed with an empty vector or a plasmid carrying respective wildtype gene (pypfP, pltaA, and ptagO), were cultured in silkworm plasma at 37°C for 7 h and aggregates were observed. B) The dry weights of the aggregates observed in C) were measured. Means±standard errors from three independent experiments are presented. Student’s t-test p-value less than 0.05 are shown (*, the gene-knockout strain vs. the parent strain; **, the complemented strain vs. the gene-knockout strain).

Based on the results that arabinose and galactose inhibited *S*. *aureus* aggregation, we hypothesized that bacterial surface polysaccharides are involved in *S*. *aureus* aggregation in silkworm plasma and examined the effect of knockout of *S*. *aureus* genes involved in teichoic acid synthesis: *ypfP*, which encodes synthetase for diglucosyl-diacylglycerol, the membrane anchor of lipoteichoic acids [27]; *ltaA*, which encodes translocase of diglucosyl-diacylglycerol from the inner to the outer leaflet of the membrane [28]; and *tagO*, which encodes synthetase for wall teichoic acids [29]. The *ypfP*, *ltaA*, and *tagO* knockout strains exhibited severely decreased aggregation in silkworm plasma compared with the parent strain (Fig 4A and 4B). The decreased aggregations of the *ypfP*, *ltaA*, and *tagO* knockout strains were respectively restored by introducing wildtype *ypfP*, *ltaA*, *tagO* genes (Fig 4A and 4B). These findings suggest that bacterial cell surface teichoic acids are required for *S*. *aureus* aggregation in silkworm plasma.

### *S*. *aureus ypfP* and *ltaA* genes are required for *S*. *aureus* virulence in silkworms

**Fig 5:**
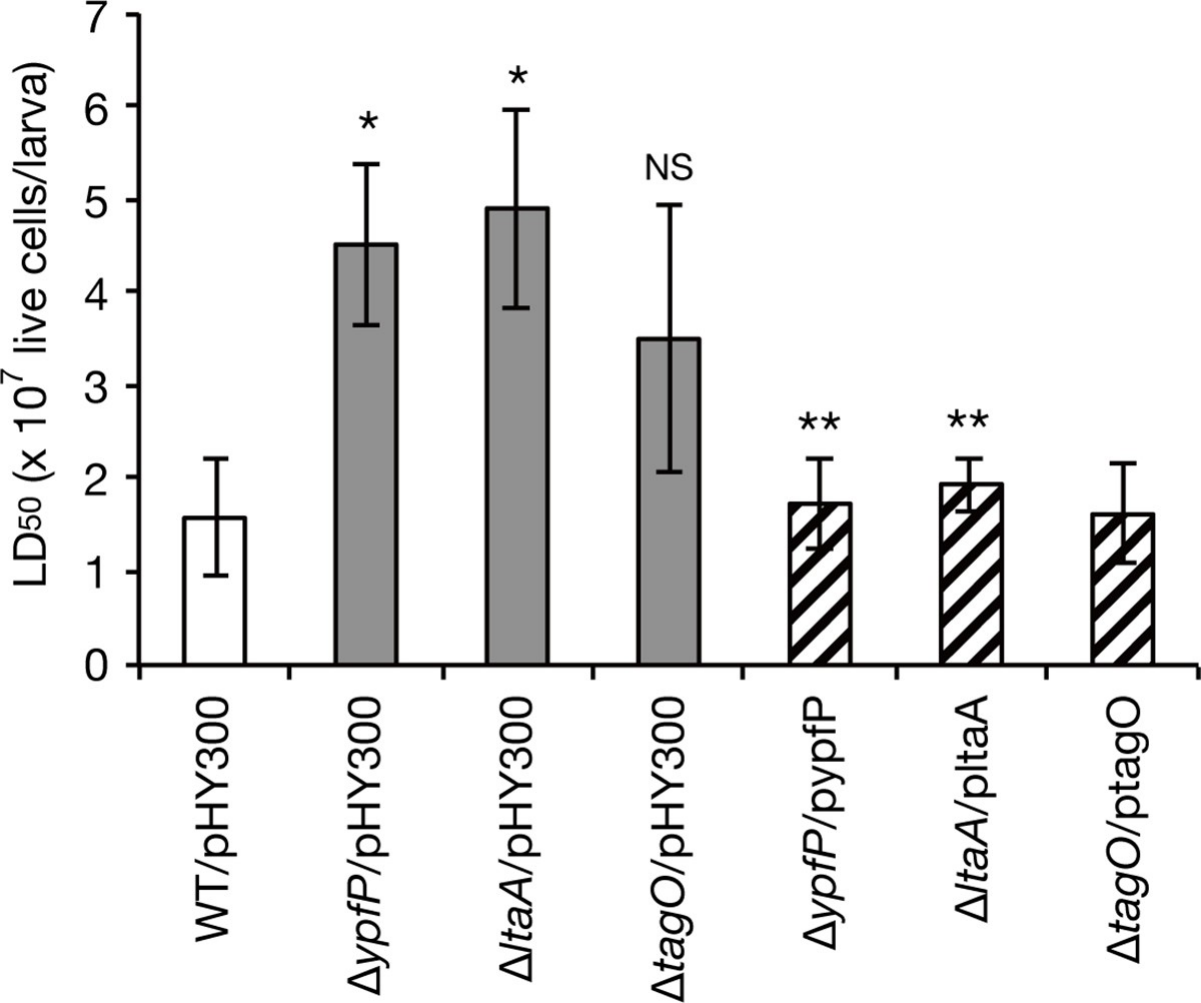
Knockout of the *ypfP* and *ltaA* genes attenuates *S*. *aureus* virulence against silkworms. Silkworms (n = 5/dose) were injected with serial dilutions of *S*. *aureus* overnight cultures of the parent strain (WT/pHY300), the knockout strains of the *ypfP*, *ltaA*, and *tagO* genes (Δ*ypfP*/pHY300, Δ*ltaA*/pHY300, Δ*tagO*/pHY300), and the complemented strains (Δ*ypfP*/pypfP, Δ*ltaA*/pltaA, Δ*tagO*/ptagO). The silkworm survival was evaluated at 24 h after the injection and the LD_50_ values were determined by logistic regression. The mean LD50 values with standard errors from three independent experiments are presented. Student’s t-test p-value less than 0.05 are shown (*, the gene-knockout strain vs. the parent strain; **, the complemented strain vs. the gene-knockout strain). NS means not significant.

To determine whether the ability of *S*. *aureus* to form aggregates in silkworm plasma is involved in *S*. *aureus* virulence against silkworms, we examined the virulence activity of *ypfP*, *ltaA*, and *tagO* knockout strains by measuring the LD_50_ value, the bacterial dose that kills half of the silkworms. The LD_50_ values of the *ypfP* and *ltaA* knockout strains against silkworms were higher than the parent strain, whereas the LD_50_ value of the *tagO* knockout strain was not (Fig 5). The LD_50_ values of the *ypfP* and *ltaA* knockout strains were restored to the level of the parent strain by introducing intact *ypfP* and *ltaA* genes, respectively (Fig 5). These findings suggest the *ypfP* and *ltaA* genes are required for *S*. *aureus* killing activity against silkworms.

## Discussion

The present study demonstrated that *S*. *aureus* forms bacterial aggregates in silkworm plasma *in vitro*, which is the first indication that *S*. *aureus* bacterial aggregates in animal plasma is not specific to mammals, but is conserved in insects.

This study revealed that *S*. *aureus* growth in silkworm plasma is required for the formation of aggregates and that the aggregation process requires the *ypfP*, *ltaA*, and *tagO* gene-encoding enzymes involved in teichoic acid synthesis. Thus, the interaction between *S*. *aureus* cell surface teichoic acids and host factor(s) in the silkworm plasma during *S*. *aureus* growth causes bacterial aggregation. Because *S*. *aureus* growth was required for the reaction, *S*. *aureus* molecule(s) specific to the logarithmic growth phase is required for the aggregation. In the coagulase reaction, which is evaluated by culturing *S*. *aureus* cells in rabbit plasma for 2–4 h, coagulase secreted from *S*. *aureus* cells activates coagulase reacting factor (CRF) in the rabbit plasma, and the activated CRF converts fibrinogen to fibrin, resulting in plasma clotting [30, 31]. Identification of such coagulase-like molecule is important to understand the molecular mechanism of *S*. *aureus* aggregation in silkworm plasma.

The coagulase reaction utilizes plasma clotting, a host immune reaction, to enable bacterial escape from humoral and cellular bactericidal reactions in the host and contributes to bacterial virulence [7, 8]. Single knockout of the coagulase gene dose not decrease *S*. *aureus* virulence in mice or rats [32–34], although double knockout of the coagulase gene and von Willebrand factor binding protein gene, another coagulation factor, decreases *S*. *aureus* virulence in mice [35]. The present study revealed that *S*. *aureus* gene knockout mutants of the *ypfP* and *ltaA* genes with decreased aggregation activity in silkworm plasma exhibit attenuated virulence in silkworms, whereas the *tagO*-knockout mutant with decreased aggregation activity does not attenuate virulence. Because the decreased aggregation activities were indistinguishable between the three mutants, it cannot conclude the involvement of the aggregation activity in *S*. *aureus* virulence in silkworms. To clarify the physiological role of *S*. *aureus* aggregation in silkworm plasma, further studies are needed such as constructing a gene-knockout mutant with severely decreased aggregation, or evaluating detailed infection processes in the silkworm model.

Nodule formation is an aggregate of insect hemocytes encapsulating invading microorganism, a cellular immune reaction in insects [36]. Carbohydrate binding proteins in the insect hemolymph called lectins, such as hemocytin and BmMBP, are involved in nodule formation [37, 38]. Because *S*. *aureus* aggregation in silkworm plasma was inhibited by the addition of monosaccharides, lectins in the silkworm plasma are presumed to be involved in the *S*. *aureus* aggregation formation. Thus, nodule formation and *S*. *aureus* aggregation both involve lectins, raising the possibility that the *S*. *aureus* aggregation in silkworm plasma is one of the aspects of nodule formation. However, there are several different features between nodule formation and the *S*. *aureus* aggregation in silkworm plasma. First, nodule formation requires insect hemocytes, whereas the *S*. *aureus* aggregation occurs in silkworm plasma lacking hemocytes. Second, *S*. *aureus* aggregation requires bacterial growth, but nodule formation does not require bacterial growth, because nodule formation occurs by formaldehyde-treated *E*. *coli* or *Micrococcus luteus* [37, 39]. Third, nodule formation is a rapid reaction that occurs within minutes after bacterial invasion [40]. When *Saccharomyces cerevisiae* or *E*. *coli* was injected into the silkworm haemocoel, transparent nodules formed within 1 min [37], and black aggregates were observed after 30 min [39]. On the other hand, *S*. *aureus* aggregation in silkworm plasma was not observed for at least 5 h. Based on these differences between nodule formation and *S*. *aureus* aggregation in silkworm plasma, we assume that *S*. *aureus* aggregates are formed *via* different molecular mechanisms than nodules.

This study unveiled a new ability of *S*. *aureus* to form bacterial aggregates in silkworm plasma and identified teichoic acids as a key factor. This finding will facilitate our understanding of *S*. *aureus* versatile ability to act not only on mammalian system but also on insect system, and enables future studies to investigate the conserved molecular mechanism of bacterial aggregation between mammals and insects.
